# The role of the pregnancy heart team in clinical practice

**DOI:** 10.3389/fcvm.2023.1135294

**Published:** 2023-04-17

**Authors:** Fabiana Lucà, Furio Colivicchi, Iris Parrini, Maria Giovanna Russo, Stefania Angela Di Fusco, Roberto Ceravolo, Carmine Riccio, Silvia Favilli, Roberta Rossini, Sandro Gelsomino, Fabrizio Oliva, Michele Massimo Gulizia

**Affiliations:** ^1^ Cardiology Department, Grande Ospedale Metropolitano, GOM, AO Bianchi Melacrino Morelli, Reggio Calabria, Italy; ^2^ Clinical and Rehabilitation Cardiology Department, San Filippo Neri Hospital, ASL Roma 1, Roma, Italy; ^3^ Cardiology Department, Mauriziano Hospital, Torino, Italy; ^4^ U.O.C. Cardiologia e UTIC Pediatrica, AORN dei Colli, Ospedale Monaldi, Università Della Campania “L. Vanvitelli”, Napoli, Italy; ^5^ Cardiology Unit, Giovanni Paolo II Hospital, Lamezia, Italy; ^6^ Cardiovascular Department, Sant'Anna e San Sebastiano Hospital, Caserta, Italy; ^7^ Department of Pediatric Cardiology, Meyer Hospital, Florence, Italy; ^8^ Cardiology Unit, Ospedale Santa Croce e Carle, Cuneo, Italy; ^9^ Cardiothoracic Department, Maastricht University Hospital, Maastricht, Netherlands; ^10^ Cardiology Unit, ASST Grande Ospedale Metropolitano Niguarda, Milano, Italy; ^11^ Cardiology Department, Garibaldi Nesima Hospital, Catania, Italy

**Keywords:** acquired heart disease, corrected congenital heart disease, pregnancy heart team, cardio obstetric team, pre-conception counseling, multidisciplinary team-Based approach, postpartum followup

## Abstract

Significant maternal and fetal morbidity and mortality risk has been shown to be associated with cardiovascular disease in pregnancy. Several determinants, such as the increasing number of females with corrected congenital heart disease in reproductive age, a more advanced maternal age associated with cardiovascular risk factors, and a greater prevalence of preexisting comorbidities related to cardiac disorders such as cancer and COVID-19), lead to a higher incidence of cardiac complications in pregnancy in the last few decades. However, adopting a multidisciplinary strategy may influence maternal and neonatal outcomes. This review aims at assessing the role of the Pregnancy Heart Team, which should ensure careful pre-pregnancy counseling, pregnancy monitoring, and delivery planning for both congenital and other cardiac or metabolic disorders, addressing several emerging aspects in the multidisciplinary team-based approach.

## Introduction

Maternal mortality (MM) has increased in the last twenty years (1). A substantial role of cardiovascular diseases (CVD) in this rising trend has been well-established (2), and more than 33% of pregnancy-related deaths have been attributed to CVD (3–6). Corrected congenital heart disease (cCHD), valvular heart diseases (VHD), and cardiomyopathies are the most frequent CVD in pregnancy (7). Moreover, because of the improvements in cCHD surgery, it has become more and more frequent that females survivors with cCHD embark on pregnancy (2, 8); in addition, a more advanced maternal age (9) and, consequently, a greater prevalence of cardiovascular (CV) risk factors have been shown to contribute to CV deaths and morbidity (2). Indeed it has been recognized that women over 30 years have a higher MM rate (9). Conversely, the development of complications such as intrauterine growth restriction (IUGR), preterm birth, preeclampsia, and other hypertensive disorder of pregnancy are more likely to be related to future CVD after delivery (10).

Remarkably, it has been estimated that more than 68% of CV-related MM could have been avoided (5). Therefore, in order to minimize MM, a multidisciplinary approach to pregnancy-associated conditions has been advocated (11, 12). In the latest decades, it has been proposed to create a cardio-obstetric or pregnancy heart team (PHT) involving cardiologists, gynecologists, obstetrics, anesthesiologists, nurses, and other specialists according to the specific clinical competencies required. Women with CVD or CV risk factors should be referred to this multidisciplinary team to improve care and outcomes for those at higher risk. The role of PHT is not only limited to the pregnancy period, but it is also crucial before pregnancy and in the post-delivery period (11–13). Indeed, women referred to PHT should receive appropriate counseling on maternal and fetal risk and the potential teratogenic effects of several drugs. Contraception should also be provided if required. An accurate clinical examination and close follow-up during pregnancy and, importantly, planning delivery should be provided. Finally, in the post-partum period, women should be carefully monitored for managing CV complications.

However, although the positive impact of team-based multidisciplinary strategies on pregnancy outcomes has been assessed, the role of the PHT has not been definitively recognized in clinical practice, and significant gaps exist in implementing a multiplanar approach for reducing pregnancy-associated comorbidity CVD burden. This paper aims to comprehensively discuss the efficacy and appropriateness of multidisciplinary evaluation, which enables an improvement in quality care.

## Epidemiology

Maternal death is defined as a non-accidental, pregnancy-related fatal event occurring during pregnancy or within 42 days of its termination, irrespective of its duration and location (14). Moreover, all life-threatening events occurring during pregnancy and delivery are defined as severe maternal morbidities.

It has been estimated that the global maternal mortality ratio (MMR) is 216/100,000 live births (1), with a wide variability between developing and more developed countries (1). Indeed, social and geographic differences have also been considered to influence the pregnancy outcome, and a significant geographical heterogeneity has been shown. Black and Hispanic ethnicities belonging have been considered pregnancy-related mortality risk factors (15). Notably, a particularly high MMR (12/100.000) has been reported in the United States (1). Conversely, a lower mortality rate was recorded in European countries (1, 16), ranging from 2.7/100.000 to 10.9/100.000 live births in Norway and Slovakia, respectively (16). It has been reported that MM occurs mostly in preterm women or at delivery. However, a prevalence of MM of 20% until six weeks postpartum and later has been reported. CVD has been shown to cause more than ¼ of MM so that they are considered one of the major causes of MM (17).

## Physiopathology

Blood volume expansion, higher cardiac output, lower systemic vascular resistance, obstruction of the vena cava, anemia, and systemic blood pressure (BP) fluctuations are hemodynamic changes that physiologically characterize each pregnancy and can result in the worsening of preexisting CVD (18). Therefore, underlying CV conditions (19) can be exacerbated by pregnancy.

Conversely, acquired CVD, can develop during pregnancy (19).

## Pregnancy heart team

Although the awareness of MMR has increased in the last decades, the management of these patients still needs to be better organized.

In order to improve the quality of care for complex pregnant women avoiding discrepancies among different hospitals, the development of a PHT including cardiologists, gynecologists, anesthesiologists, and other specialized figures such as geneticists, neonatologists, cardiac surgeons, endocrinologists, and oncologists has been proposed (13, 17, 20, 21). Teams has been proposed. PHT should be finalized not only to guarantee accurate monitoring during the pregnancy and delivery but also should be organized to last from pre-conception counseling (22) to the postpartum follow-up, including labor and delivery time (14, 17–19).

## Pre-conceptional counseling

Pre-conceptional counseling before pregnancy is crucial for identifying high-risk patients for maternal and fetal complications (22, 23). Contraception in young patients with CVD should also be encouraged by the PHT in order to avoid unplanned pregnancies (22). Moreover, women should be supported by the PHT and provided with the opportunity to choose the best timing for the pregnancy and undergo planned treatment. Remarkably, the inheritance of several conditions should also be faced. Heredity is expected to manifest in 50% of patients with genetic disorders associated with cCHD, such as DiGeorge (22q11 deletion) (24, 25), Marfan (26), Heart-hand syndromes (26, 27) Holt-Oram (28), Nooman (29), Alagille (30), CHARGE (31), Williams-Beuren (32), Cutis laxa (33), Vascular Ehlers–Danlos (vED) (34), and Silver-Russel syndromes (35). Several scores have been proposed to assess risk in pregnant women or those planning pregnancy. CARPREG II (Cardiac Disease in PregnancyStudy) (36), ZAHARA (Zwangerschap Bij Aangeboren Hartafwijking) (37), and modified WHO (World Health Organization) (38–40) have been validated to clinically evaluate the CVD burden, in order to favor not only preconception counseling, but also pregnancy and delivery management, and eventually termination of pregnancy in particularly high-risk conditions.

The modified World Health Organization (mWHO) risk score identifies five risk classes (WHO I, II, II-III, III, and IV) (38–40), investigating not only the risk assessment of CV events but also obstetric complications, such as miscarriage, postpartum hemorrhage, hypertensive disorders, prematurity, intrauterine growth restriction (IUGR), low birth weight (ELBW), and perinatal mortality (38).

Preconception counseling, frequency of controls during pregnancy, delivery time and modality, and postpartum care should be based on the risk assessment (8, 41–47).

## Postpartum follow-up

Postpartum follow-up should be adequately monitored in women with known CVD, considering the fact that women with CVD remain at high risk for late CV complications (48). Females with an increased risk for adverse long-term CV outcomes can be identified using pregnancy risk prediction tools (48). The intrauterine device or progesterone-only subdermal implants can be used in the immediate postpartum period, taking into account the risk of thrombosis or bleeding.

Patients at great risk for developing CV complications should be monitored for the following 72 h (49). Several CV events, such as peripartum cardiomyopathy (PPCM), pulmonary embolism (PE), spontaneous coronary artery dissection (SCAD), and aortic dissection (AD), can occur postpartum. The so-called “red flag” symptoms are thought to be relevant in the early detection of CV complications. A self-monitoring of the patients is also beneficial. After discharge, the first visit should be performed for high-risk patients within three days (50). A postpartum evaluation within the first three weeks after delivery with interim follow-up has been recommended, including a comprehensive medical examination within 12 weeks (50). Nevertheless, heart failure (HF), arrhythmias, hypertensive disorders, and hemorrhagic and infective pregnancy-related events complications have been considered the most frequent causes of rehospitalizations in the first 42 days postpartum (51).

## Discussion

An increment in MM has been reported in the last two decades. CVD is considered the leading cause of MM and morbidity (3–6). Nowadays, advanced maternal age is commonly observed, being related to a more prevalence of comorbidities and CV risk factors such as hypertension, diabetes, and obesity (15).

In addition, the widespread use of assisted reproductive technology has been correlated with greater CV risk. On the other hand, the improved management of cCHD resulted in a more prevalence of adult females with cCHD. Due to the difficult management of CVD in pregnant women, referring these patients to highly specialized centers would be advisable to ensure a high quality of care and a multidisciplinary approach during pregnancy and in the first few months of postpartum (Table 1).

**Table 1:** Role of PHT evaluation before, during, and after pregnancy in the most common CVD.

CVD	Before conception	During peripartum	Delivery	Long term follow-up
**CAD (15, 18, 38, 52)**	– History of CAD – Evaluation of ongoing medications	– Assessment for possible ACS: symptoms, ECG, echocardiography – Evaluation of CAG indication – Management for antiplatelet therapy: (aspirin, clopidogrel for shortest duration) – Medications (beta-blockers, nitrates)	– Prefer vaginal delivery – Correct anemia and volume depletion (may exacerbate underlying ischemia) – Avoid hypotension/hypertension – Avoid bleeding and arrhythmias	– Contraception – Counseling for the evaluation of future pregnancies
**Cardiomyopathies (38, 40)**	– History of cardiomyopathy – Baseline echocardiogram – BNP/NTproBNP – Functional class classification – Evaluation of medications safety	– Acute HF management – Echocardiographic FU – Evaluation of medications’ safety during pregnancy and postpartum – Avoiding hypotension and excessive diuresis – Anticoagulation in women with PPCM	– Based on hemodynamic conditions and choice of team – Monitor for 72 h after delivery	– Considering FU within 7–10 days. – Considering anticoagulation for 6–8 weeks if LVEF < 35% in women with PPCM – Contraception
**Hypertrophic cardiomyopathy (38)**	– Echocardiographic evaluation – Avoiding pregnancy if severe LV dysfunction or severe symptomatic LVOTO occur	– Evaluation of medications safety: beta-blockers and calcium channel – Multidisciplinary clinical and echocardiographic approach (every 3 months or in case of hypotension)	– Vaginal delivery is preferred	– Close monitoring for volume depletion (blood loss may worsen LVOTO)
**Arrhythmias (38, 40)**	– History of arrhythmias – Devices – Drug evaluation: antiarrhythmics and anticoagulants	– Acute treatment of arrhythmias – Multidisciplinary approach – VA: amiodarone o synchronized ECV in case of hemodynamic instability – SVA: vagal maneuvers, if adenosine is not effective – Medical therapy: antiarrhythmics and anticoagulants – Zero x-ray CA may be considered in selected patients with frequent recurrences despite medical therapy	– Vaginal delivery is preferred	– Medical therapy: antiarrhythmics and anticoagulants – Consider CA
**VHD (18, 38)**	– Clinical and echocardiographic assessment – -Severe VHD should be treated before conception – -Consider valve repair or bioprosthetic valve replacement in order to minimize the need for anticoagulation	– Clinical and echocardiographic assessment	PHT for deciding mode and timing of delivery	– Clinical and echocardiographic assessment
**Mitral stenosis (18, 38)**	– Clinical and echocardiographic assessment – Valvuloplasty if the valve area is ≤1 cm^2^	– Clinical and echocardiographic assessment – Valvuloplasty in patients with symptoms or pulmonary hypertension (sPAP) > 50 mmHg) under OMT – Treatment of HF	– Vaginal delivery is preferred – Caesarean section is generally considered in patients in NYHA class III/IV or with PHA	– Regular FU visits after delivery – Late prognosis depends mainly on stenosis progression. – Regular FU are required
**Aortic stenosis (18, 38)**	– Clinical and echocardiographic assessment – Consider reparative therapy – Pregnancy is generally well tolerated in mild to moderate AS	– Clinical and echocardiographic FU every two months – Acute HF management – Severe AS symptomatic despite medical therapy, percutaneous treatment shoul be considered	– Vaginal delivery is preferred in non-severe aortic stenosis – Caesarean delivery should be preferred in severe symptomatic AS	– Evaluation of AS degree – Disease progression is frequent after delivery – Close FU are required
**Pulmonary artery hypertension (38)**	– Consider echocardiography and right heart catheterization – It is confirmed PAH, pregnancy should be avoided – When pregnancy occurs, termination should be evaluated	– Treatment of pulmonary hypertensive crisis, thrombosis, and right HF	– PHT for the decision on the mode and timing of delivery	Counseling is necessary to discuss the need for ongoing therapies and to avoid future pregnancies
**Atrial Septal Defect (38)**	– Consider Surgical or Percutaneous ASD closure – Echocardiographic evaluation	– In unrepaired defect, treat thromboembolic complications and atrial arrhythmias	– Vaginal delivery is preferred	– Closure should be considered
**Coarctation of the aorta (19, 38)**	Consider repair	– Close BP monitoring for unrepaired CoA – Echocardiographic FU: aneurysms have an increased risk of complications, including dissection	– Vaginal delivery is preferred – Close BP monitoring	– Close BP monitoring
**Fontan circulation (19, 38)**	– Clinical and echocardiogram assessment – Pregnancy shoul be discouraged	– Frequent surveillance during pregnancy (monthly) – Consider anticoagulation for thromboembolic complications – Treat arrhythmias promptly	– The time and modality of the delivery should be programmed by PHT	– Surveillance in the first weeks after delivery
**Tetralogy of Fallot (19, 38)**	– Maternal screening for 22q11 deletion	– Clinical evaluation every three months – Treatment with diuretics and bed rest if right ventricular dysfunction develops	– PHT for deciding mode and timing of delivery	Close surveillance
**Aortic Disease (38)** **Marfan syndrome** **Vascular Ehlers–Danlos syndrome** **Turner syndrome**	– Genetic counseling – Counselling that evaluates the risks of aortic dissection (aortic dilation) – Imaging of the entire aorta (CT/MRI) – Pregnancy is not recommended in Vascular Ehlers-Danlos syndrome and Turner syndrome with severe dilatation of the aorta	Monitoring by echocardiography every month in high risk patients and every three months in low-risk patients – Strict BP control (prefer beta-blockers) – Aortic dissection occurring during pregnancy is a surgical emergency – Multidisciplinary team (cardiothoracic, cardiology, obstetric, and cardio-anesthetic physicians) must act rapidly to deliver the fetus by cesarean section in specialized cardiothoracic centers and promptly repair the dissection. If pregnancy is not viable, aortic surgery with the fetus in place should be performed	Vaginal delivery if the ascending aorta diameter is <45 mm Cesarean delivery should be considered when the aortic diameter exceeds 45 mm, and is recommended in patients with vascular Ehlers–Danlos syndrome type IV	FU of the dilated aorta
**Hypertension (15, 38, 40, 52)**	– History of chronic hypertension – Antihypertensive therapy – Prevention of eclampsia with low-dose aspirin – Chronic hypertension includes evaluation of target organ involvement and evaluation of secondary causes	– Modification of diet and lifestyle – Treatment of moderate to severe and acute hypertension (labetalol, alpha-methyldopa and calcium channel blockers as first-line therapy)	– Vaginal delivery with close BP control	– Adjustment of postpartum therapy – Monitoring BP – Postpartum BP monitoring is recommended within 72 h and no later than ten days after hospital discharge

CAD, coronary artery disease; ACS, acute coronary syndrome; ECG, electrocardiogram; CAG, coronary angiography; FU, follow-up; BNP, brain natriuretic peptide; NT-proBNP, N-terminal (NT)-pro hormone BNP; HF, heart failure; PPCM, peripartum cardiomyopathy; LVEF, left ventricular ejection fraction; LV, left ventricular; LVTO, left ventricular outflow tract obstruction; ECV, electrical cardioversion; VA, ventricular arrhythmias; CA, catheter ablation; sPAP, systolic pulmonary artery pressure; OMT, optical medical therapy; PAH, pulmonary arterial hypertension; MS, mitral stenosis; AS, aortic stenosis; ASD, atrial septal defect; BP, blood pressure; CoA, coarctation of the aorta.

### Heart failure

Pregnancy-related HF is a dangerous condition that requires an appropriate multidisciplinary approach. PPCM (53, 54) and pre-existing CVD (36, 55) have been reported to be the leading causes of HF development during pregnancy. However, diastolic dysfunction may also evolve in overt HF (56, 57). Therefore, if clinical signs and/or symptoms occur, echocardiographic parameters and biomarkers should be strictly monitored in order to detect HF early (11).

Women may become symptomatic for HF in the second trimester or earlier due to increased plasma volume, especially if structural cardiac disorders coexist (58). Nevertheless, it has been reported that 60% of pregnancy-related HF occurs postpartum, particularly in the 30 days following delivery (59). However, the diagnosis is frequently delayed or under-recognized.

Beta-blockers (except atenolol), thiazides, and loop-diuretics should be recommended, whereas angiotensin-converting enzyme (ACE) inhibitors/angiotensin II receptor blockers (ARBs), mineralocorticoid receptor antagonists, and angiotensin receptor-neprilysin inhibitor (ARNI) should not be used due to their fetotoxicity. Moreover, diuretics use should be limited to those cases in which pulmonary congestion (60). Hydralazine and nitrates may be safely used during pregnancy (61, 62). The delivery option should be evaluated if an acute refractory HF is detected. Sodium restriction should be recommended for all patients.

### Peripartum cardiomyopathy (PPCM)

PPCM may occur during pregnancy or after delivery, generally in the earlier phases with idiopathic etiology. Its incidence ranges from 1 to 100 and 1–60,000 live births (63–67). African-American (AA) ancestry, a more advanced maternal age, multiple pregnancies, genetic predisposition, and hypertensive disorders have correlated with PPCM (63, 64, 68–70).

PPCM has been reported to be a leading cause of MM (71, 72). The diagnosis may be challenging because signs and symptoms may be masked by normal late pregnancy and postpartum features. A delay in detecting the diagnosis significatively increases MM so that an early diagnosis is crucial. Remarkably, PHT should provide the most appropriate medical strategy, carefully evaluating the potential teratogenic drugs effect and balancing advantages and drawbacks for the mother and fetus. Beta-blockers, loop diuretics, hydralazine/isosorbide dinitrate, and digoxin use may be encouraged, whereas ACE/ARB/aldosterone receptors antagonists must not be used. Moreover, to avoid thromboembolic events, anticoagulation should be considered during pregnancy in patients with LVEF <40%, prolonging to the first eight weeks after delivery (73).

Other pharmacological approaches, such as intravenous immune globulin use (74), pentoxifylline (an anti–tumor necrosis factor-alpha) (75), and bromocriptine prolactin inhibitor (76, 77) have also been proposed. After delivery, enalapril and spironolactone may be initiated, as well as beta-blockers and diuretics may be continued, preventing patients from fluid overload. Furthermore, if a severe LV dysfunction persists, PHT should consider wearable cardioverter/defibrillator options. Long-term follow-up also is recommended. Finally, contraception options should be guaranteed.

### Coronary artery disease

Acute myocardial infarction associated with pregnancy (PAMI) has been shown to have a 3-fold increased prevalence in pregnancy compared to what has been expected in women of similar age and CV comorbidities (78), with a reported incidence of 1/16,000 deliveries (79). Pregnant women of all ages can be affected, particularly those aged more than 30 years (78). In addition to the traditional risk factors, other predisposing conditions, such as pre-eclampsia and eclampsia, have been described (79).

SCAD is the leading cause of PAMI, especially in the latest gestational period and in the early post-partum. The left anterior descending artery and left main segment are the most commonly involved vessels (80). Structural hormonally-mediated coronary alterations belonging to the hypercoagulable state of pregnancy have been proposed as PAMI-related mechanisms of coronary thrombosis in the absence of atherosclerosis. Transient spasms may also underline a SCAD if normal coronary artery anatomy is found (81).

ST-segment elevation myocardial infarction (STEMI) is the most common clinical manifestation of PAMI. LV function impairment and ventricular arrhythmias (VA) may occur (80). Due to very high mortality (ranging from 5% and 7%) in both mother and fetus (80, 82), PHT evaluation is crucial. A percutaneous coronary intervention should be recommended regardless of pregnancy. However, radiation risks must be carefully taken into account, lowering fetal exposure in order not to exceed the cutoff (<1 rad during pregnancy) (83). Moreover, the increased risk of SCAD should be considered.

Conversely, a conservative approach should be evaluated in non-ST-elevation myocardial infarction (NSTEMI) (80). Remarkably, the PHT approach in this context is mandatory.

### Congenital heart disease (CHD)

Due to the improvement in cardiac surgery that has raised congenital patients' survival (84), the percentage of pregnant women with cCHD requiring a PHT evaluation has increased in the last decades. Although MM has been dramatically lowered up to 0.5% (47), cCHD causes a significant morbidity burden, often resulting in arrhythmias and HF (55, 85–88), so that strict clinical follow-up should be performed. Moreover, CHD must be classified into subcategories, accurately assessing the pregnancy-related risk according to the mWHO risk score (38–40). PHT plays a crucial role in managing these patients, who must be provided with appropriate counseling to raise awareness of pregnancy-related risks (89).

Remarkably, also according to the European Society of Cardiology (ESC) (18, 19, 40), Fontan circulation, systemic right ventricle (RV), and uncorrected cyanotic CHD are considered high-risk congenital disorders which mostly need a PHT evaluation.

### Metabolic disorders

Metabolic disorders such as gestational diabetes mellitus should be detected and treated the earliest as possible (23), due to potential complications and adverse long-term consequences for both mother and fetus (90). Therefore, identifying undiagnosed prediabetes or diabetes at the beginning of the pregnancy is essential to improve pregnancy outcomes (91).

### Pulmonary arterial hypertension (PAH)

Pulmonary Arterial Hypertension (PAH) is likely to be due to a multifactorial etiology. Idiopathic or heritable etiology, as well as connective tissue disease, CHD (Eisenmenger syndrome), left heart disorders, pulmonary diseases, and thromboembolic diseases, have been reported as mechanisms for the development of PAH (92). Patients with PAH must be carefully evaluated in order to be provided with the most appropriate treatment. Moreover, delivery must be planned early delivery. Remarkably, counseling is crucial for women with known PAH to decide strategies, including targeted therapies, physical exercise, oxygen support, and whether to interrupt pregnancy.

### Valvular heart disease (VHD)

Congenital and acquired VHD are important causes of MM and morbidity, despite the fact that rheumatic etiology has diminished in the last decades, remaining a leading cause in developing countries (40, 93, 94). Remarkably, mechanical prosthetic valve management in pregnancy is particularly complex, requiring an appropriate anticoagulation strategy, requiring a PHT-based approach before and during pregnancy (40, 93, 94).

### Cancer

Notably, due to the rising prevalence of cancer at young ages, the number of survivors who reach reproductive age and desire a pregnancy is significantly increased (95). In these cases, it is crucial that the patient is aware of the influence of cancer-related treatments on fertility, the outcome of the pregnancy, and potential CV complications (96).

It has been recognized that LV dysfunction may develop in women survivors who have undergone cancer therapies at reproductive age (37). Moreover, pregnancy-related hemodynamic stress is likely to result in LV impairment and HF (97).

The main risk factors of CV events during pregnancy include a reduced LV systolic function prior to the pregnancy, history of chemotherapy with anthracyclines (cumulative dose of doxorubicin ≥ 250 mg/m^2^) (98), history of radiotherapy (cumulative dose ≥ 35 Gy or direct radiation on the heart > 15 Gy), diagnosis and treatment of cancer at a young age (<10 years), a longer period of time from cancer treatment to first pregnancy (>15 years) (99, 100). The assessment of the basal BNP value during pregnancy allows early identification of systolic function impairment (101, 102). Moreover, women with a history of cardiomyopathy are at a higher risk of developing further LV failure during pregnancy (103).

Notably, late radiation-induced complications may occur after radiotherapy, manifesting as therapeutics-related cardiac dysfunction, premature CAD, valvular abnormalities, pericardial injury, HF, pericardial disease, and arrhythmias (104).

Therefore, it has been established that cancer survivors who are planning a pregnancy should undergo pre-conceptional counseling (100). Clinic surveillance, including echocardiographic evaluation, is advisable before pregnancy for patients previously treated with anthracyclines and chest radiation (100).

### COVID-19

An increment of 33% in MMR during the COVID-19 pandemic has been reported (105).

It has been recognized that COVID-19 patients have associated injury of the heart and vessels involving microvascular and macrovascular damage. Arterial and venous thromboembolism, CAD, HF, and arrhythmias have been shown to increase in COVID-19. Pregnant women, compared to non-pregnant females affected by COVID-19 disease, are more likely to have adverse outcomes. Moreover, severe infections (10%), intensive care unit (ICU) admission (4%), mechanical ventilation (3%), and extracorporeal membrane oxygenation (ECMO) needing (0.2%) have been reported to be more frequent in COVID-pregnant patients (106). Furthermore, COVID-19 complications may lead to preterm delivery, and the management of the pregnancy is substantially modified (107).

Assessing pregnant women with COVID-19 requires PHT to recognize COVID-19-related CV complications and to distinguish them from other pregnancy-related CV risk conditions (107). Notably, a more advanced maternal age, obesity, hypertension, and diabetes not only result in increasing CV risk in pregnancy but also the risk of severe COVID-19 disease. Accordingly, a higher neonatal ICU rate has been recorded in children of mothers affected by COVID-19. The increased risk of CV complications has been associated with a low vaccination rate in pregnant women. A more adverse outcome has been reported in unvaccinated women compared to vaccinated ones. Remarkably, vaccination during pregnancy should be strongly encouraged and should be included in the PHT program (108).

## Conclusions

Progress in cardiovascular care and cardiac surgery has determined significant improvement in the conditions of women who choose to become pregnant. A close assessment before the pregnancy and monitoring during and after by a multidisciplinary group is able to reduce adverse events and improve maternal-fetal outcomes.

**Figure 1:**
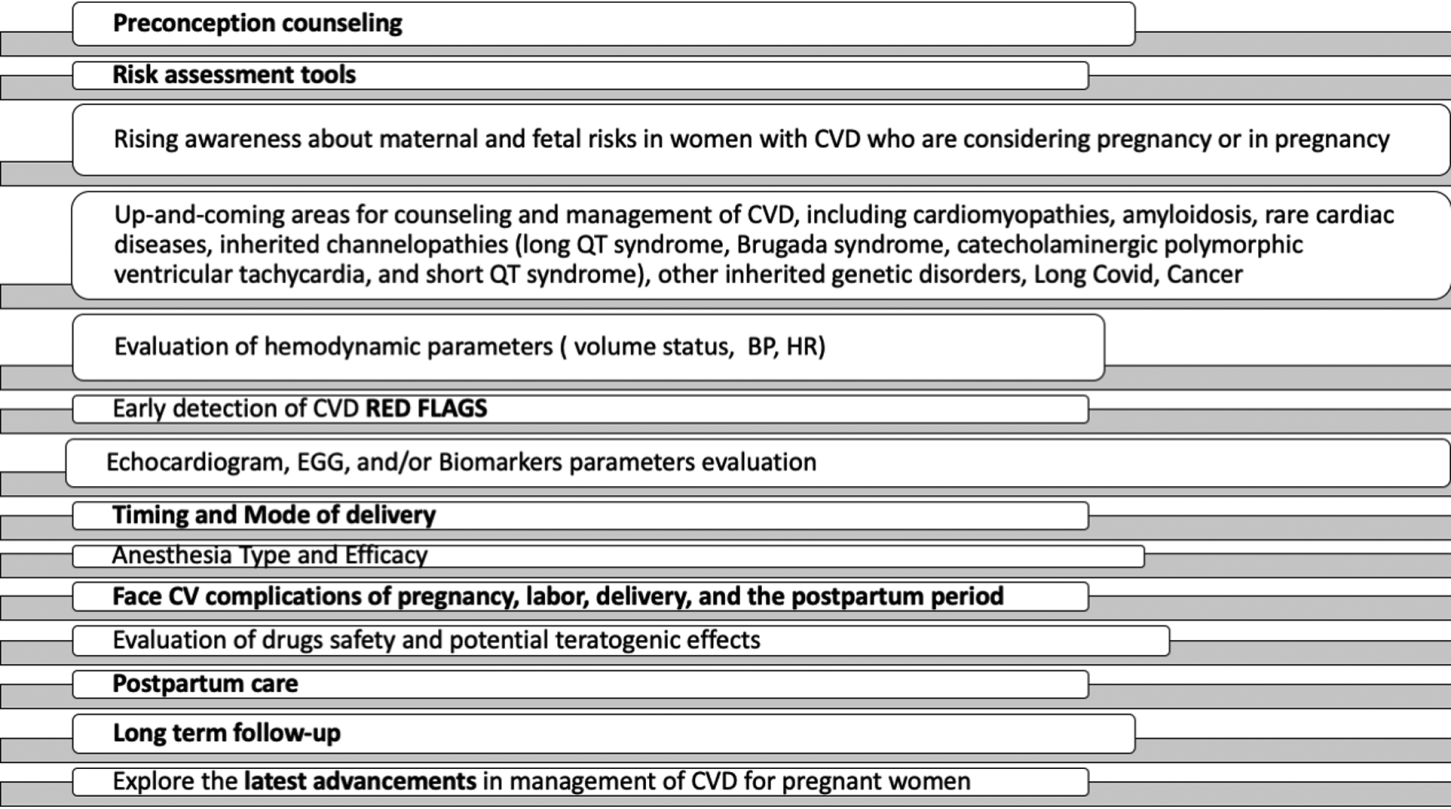
Pregnancy heart team: present and future directions. CVD, cardiovascular diseases; BP, blood pressure; HR, heart rate.

PHT management of comorbidities should be incorporated into pregnancy care in order to optimize appropriate and effective therapies (Figure 1).

Implementing PHT care will require a multidisciplinary team to address therapeutic optimization, active comorbid disease management, and evidence-based interventions. Therefore, optimizing care pathways in cardio-obstetric patients is a promising area of care innovation that should substitute the traditional care approaches.

## Author contributions

All authors have seen and approved the manuscript being submitted, have contributed significantly, attest to the validity and legitimacy of the data and its interpretation, and agree to its submission. All authors agree with the content, and all give explicit consent to submit. All authors whose names appear on the submission: 1. Made substantial contributions to the conception, and design of the work and to acquisition, analysis, or interpretation of data. 2. Drafted the work or revised it critically for important intellectual content; 3. approved the version to be published; 4. agree to be accountable for all aspects of the work in ensuring that questions related to the accuracy or integrity of any part of the work are appropriately investigated and resolved.

## Group members of Management and Quality Working Group

Fabiana Lucà (MD, PhD, FESC) (Chairperson), Giorgio Caretta Ospedale S. Andrea - La Spezia, Stefano Cornara P.O. Levante Ospedale San Paolo - Savona, Irene Di Matteo ASST Ospedale Metropolitano Niguarda - Milano, Concetta Di Nora AOU Santa Maria della Misericordia - Udine, Silvia Favilli Azienda Ospedaliero Universitaria Meyer - Firenze, Simona Giubilato Azienda Ospedaliera Cannizzaro - Catania, Anna Pilleri ARNAS G. Brotzu - Cagliari, Andrea Pozzi ASST Papa Giovanni XXIII - Bergamo and Roberta Rossini Azienda Ospedaliera Santa Croce e Carle - Cuneo.

## Group members of Pediatric Cardiology Group

Maria Giovanna Russo (MD) (Chairperson), Gabriele Egidy Assenza Ospedale Sant'Orsola Malpighi - Bologna, Annalisa Alaimo P.O. Giovanni di Cristina – Palermo, Roberta Ancona AORN Ospedale dei Colli P.O. Monaldi – Napoli, Domenico Sirico Università di Padova, Gaia Spaziani A.O.U. Meyer – Firenze, Stefano Domenicucci Agenzia Ligure Sanità Regione Liguria – Genova, Giovanni Di Salvo Azienda Ospedale-Università di Padova and Maria Giulia Gagliardi Ospedale Pediatrico Bambino Gesù – Roma.

## Conflict of interest

The authors declare that the research was conducted in the absence of any commercial or financial relationships that could be construed as a potential conflict of interest.
